# Supplementary material for: Cytotoxic T-cells mediate exercise-induced reductions in tumor growth
Source: eLife. 2020 Oct 23;9:e59996. doi: 10.7554/eLife.59996 (PMC7584454; doi:10.7554/eLife.59996)
Supplement: Transparent reporting form [file elife-59996-transrepform.docx]

***eLife’s* transparent reporting form**

We encourage authors to provide detailed information *within their submission* to facilitate the interpretation and replication of experiments. Authors can upload supporting documentation to indicate the use of appropriate reporting guidelines for health-related research (see [EQUATOR Network](http://www.equator-network.org/%20)), life science research (see the [BioSharing Information Resource](https://biosharing.org/)), or the [ARRIVE guidelines](http://www.plosbiology.org/article/info:doi/10.1371/journal.pbio.1000412) for reporting work involving animal research. Where applicable, authors should refer to any relevant reporting standards documents in this form.

If you have any questions, please consult our Journal Policies and/or contact us: [editorial@elifesciences.org](mailto:editorial@elifesciences.org).

**Sample-size estimation**

- You should state whether an appropriate sample size was computed when the study was being designed
- You should state the statistical method of sample size computation and any required assumptions
- If no explicit power analysis was used, you should describe how you decided what sample (replicate) size (number) to use

Please outline where this information can be found within the submission (e.g., sections or figure legends), or explain why this information doesn’t apply to your submission:

For all animal experiments, concurrent with experimental design, pilot experiments of N=5-6 per group were done and power calculations to estimate the required sample size for the primary outcome was performed using the G*power analysis software, with a power of 80% and a significance level of 0.05.

**Replicates**

- You should report how often each experiment was performed
- You should include a definition of biological versus technical replication
- The data obtained should be provided and sufficient information should be provided to indicate the number of independent biological and/or technical replicates
- If you encountered any outliers, you should describe how these were handled
- Criteria for exclusion/inclusion of data should be clearly stated
- High-throughput sequence data should be uploaded before submission, with a private link for reviewers provided (these are available from both GEO and ArrayExpress)

Please outline where this information can be found within the submission (e.g., sections or figure legends), or explain why this information doesn’t apply to your submission:

Information about N is found embedded in the figures or stated in figure legends. All N are biological repeats (for animals experiments N=individual animals and for ex vivo and/or in vitro experiments N= cells from individual animals and/or consecutive experiments). Technical repeats are used as quality controls within an individual experiment but are not used as the basis for variation reports or statistics. The majority of our figures includes presentation of the data as scatterplots. Outliers were identified using Grubb´s test in the GraphPad Prism software and excluded when it created a relationship were there otherwise wasn´t one. Exclusion based on “a priori” determined running distance was made: in the exercise experiments for figure 1, animals running less than 1km/day was excluded from the analysis (this was rare). For the adoptive transfer experiments in figure 5, a cut off of 6 km per night for the exercise animal group was done, to achieve per protocol analysis.

**Statistical reporting**

- Statistical analysis methods should be described and justified
- Raw data should be presented in figures whenever informative to do so (typically when N per group is less than 10)
- For each experiment, you should identify the statistical tests used, exact values of N, definitions of center, methods of multiple test correction, and dispersion and precision measures (e.g., mean, median, SD, SEM, confidence intervals; and, for the major substantive results, a measure of effect size (e.g., Pearson's r, Cohen's d)
- Report exact p-values wherever possible alongside the summary statistics and 95% confidence intervals. These should be reported for all key questions and not only when the p-value is less than 0.05.

Please outline where this information can be found within the submission (e.g., sections or figure legends), or explain why this information doesn’t apply to your submission:

Data presentation format and statistical analysis method is described in the figure legends for each figure panel and in the statistics section of methods. Analysis of data from the Precision Metabolomics platform was executed as described in the methods section under “UHPLC/MS analysis”. Throughout the ms, scatterplots are used to provide information on raw data distribution when relevant. P-values are reported as strict n.s, P<0.05, p<0.01 or p<0.001.

(For large datasets, or papers with a very large number of statistical tests, you may upload a single table file with tests, Ns, etc., with reference to sections in the manuscript.)

**Group allocation**

- Indicate how samples were allocated into experimental groups (in the case of clinical studies, please specify allocation to treatment method); if randomization was used, please also state if restricted randomization was applied
- Indicate if masking was used during group allocation, data collection and/or data analysis

Please outline where this information can be found within the submission (e.g., sections or figure legends), or explain why this information doesn’t apply to your submission:

With the exercise interventions, animals are allocated at random to the groups. The researcher executing the intervention trials is for natural reasons not blinded to group allocations. Upon sample harvest, each sample is given a study specific name not revealing group allocation, providing some level of blinding for the researcher executing the ex vivo characterizations. A similar approach was used for the samples sent for metabolomic (mass spec) analysis, all samples were given a study ID not revealing group allocation prior to sending the data and the key for group allocation was provided once data was retrieved. Exceptions were made for running distance, in the exercise experiments for figure 1, animals running less than 1km/day was excluded from the analysis (this was rare). For the adoptive transfer experiments for figure 5, a cut off of 6 km per night for the exercise animal group was done, to achieve per protocol analysis.

**Additional data files (“source data”)**

- We encourage you to upload relevant additional data files, such as numerical data that are represented as a graph in a figure, or as a summary table
- Where provided, these should be in the most useful format, and they can be uploaded as “Source data” files linked to a main figure or table
- Include model definition files including the full list of parameters used
- Include code used for data analysis (e.g., R, MatLab)
- Avoid stating that data files are “available upon request”

Please indicate the figures or tables for which source data files have been provided:

Source data files (xls) are provided for figures 2 and 4.
